# P-611. Improving Rabies vaccination delivery and access: A Quality Improvement Project

**DOI:** 10.1093/ofid/ofae631.809

**Published:** 2025-01-29

**Authors:** Ankhi Dutta, Margaret Taylor, Catherine Foster, Galit Holzmann-Pazgal, Megan James, Valerie Morgera, Michelle Velasco, Holly Oliver

**Affiliations:** Baylor College of Medicine, Houston, Texas; Baylor College of Medicine, Houston, Texas; Baylor College of Medicine, Houston, Texas; Baylor College of Medicine, Houston, Texas; Texas Children's Hospital, The Woodlands, Texas; Texas Childrens Hospital, Houston, Texas; Texas Childrens Hospital, Houston, Texas; Baylor College of Medicine, Houston, Texas

## Abstract

**Background:**

Rabies remains a global public health concern.Barriers affecting rabies post exposure prophylaxis include lack of access to the rabies vaccine, coordination of care, and costs. Due to the challenges of vaccine access and coordination, most rabies vaccines are given in Emergency Centers (ECs). Barriers faced by patients in the EC include increased wait times, costs, and lack of appropriate referral or follow up coordination. We initiated a quality improvement project (QIP) to improve access to rabies vaccination and delivery of appropriate doses of the vaccine in the Infectious Diseases (ID) Clinic at our institution.

Rabies post exposure prophylaxis in infectious diseases clinic

**Figure:**
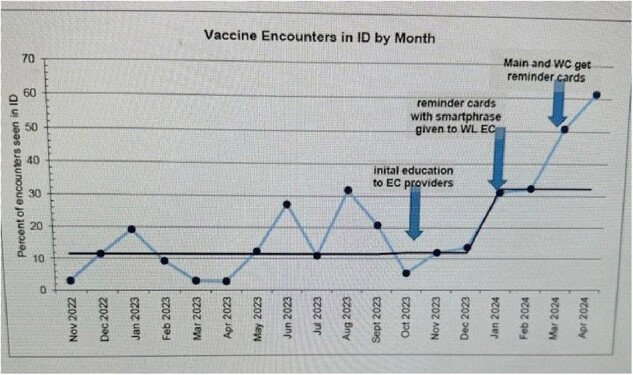

**Methods:**

Our outcome measure was to increase the total number of patient encounters for rabies vaccination in the ID Clinic across the 3 campuses at TCH by 25% in 3 months. Process measures were 1) Increase the number of referrals placed to ID by EC on first encounter by 25% in 3 months. 2) Increase the percentage of patients seen in ID clinic for all subsequent doses. Balance measure was collecting surveys to evaluate patient satisfaction with the vaccine process, including any perceived barriers. Our PDSA cycles included: 1) Education on the process to the EC providers at each campus, pharmacy, and nursing leads 2) Creating an Epic smart phrase to include the rabies vaccine schedule with appropriate dates, ID clinic phone numbers, and rabies vaccine education for families. 3) Providing small cards with the Epic smart phrase and ID referral reminder to attach to EC provider workstations at each campus. 4) Request for a standardized order set to be built in Epic to include orders for HRIG (as indicated), rabies vaccine, and referral to ID.

**Results:**

Figure 1 represents the percentage of vaccine encounters seen in the ID clinic with interventions marked. We had excellent patient satisfaction scores in the ID clinic related to less wait times, easy access and costs. There was a significant cost differential between the EC and ID clinic.

**Conclusion:**

Rabies Vaccine encounters increased from 16% to 61% between September 2023 through April 2024 across all three campuses of the hospital. Vaccines were provided with appropriate indications. Patient satisfaction was high with significant cost savings. There are currently ongoing PDSA cycles to improve access to rabies vaccine further.

**Disclosures:**

**All Authors**: No reported disclosures

